# Reverse β-oxidation pathways for efficient chemical production

**DOI:** 10.1093/jimb/kuac003

**Published:** 2022-02-26

**Authors:** Katia Tarasava, Seung Hwan Lee, Jing Chen, Michael Köpke, Michael C Jewett, Ramon Gonzalez

**Affiliations:** Department of Chemical, Biological, and Materials Engineering, University of South Florida, 4202 E. Fowler Ave., Tampa, FL 33620, USA; Department of Chemical, Biological, and Materials Engineering, University of South Florida, 4202 E. Fowler Ave., Tampa, FL 33620, USA; Department of Chemical, Biological, and Materials Engineering, University of South Florida, 4202 E. Fowler Ave., Tampa, FL 33620, USA; LanzaTech Inc., Skokie, IL 60077, USA; Department of Chemical and Biological Engineering and Center for Synthetic Biology, Northwestern University, Evanston, IL 60208, USA; Department of Chemical, Biological, and Materials Engineering, University of South Florida, 4202 E. Fowler Ave., Tampa, FL 33620, USA

**Keywords:** Reverse β-oxidation, Metabolic engineering

## Abstract

Microbial production of fuels, chemicals, and materials has the potential to reduce greenhouse gas emissions and contribute to a sustainable bioeconomy. While synthetic biology allows readjusting of native metabolic pathways for the synthesis of desired products, often these native pathways do not support maximum efficiency and are affected by complex regulatory mechanisms. A synthetic or engineered pathway that allows modular synthesis of versatile bioproducts with minimal enzyme requirement and regulation while achieving high carbon and energy efficiency could be an alternative solution to address these issues. The reverse β-oxidation (rBOX) pathways enable iterative non-decarboxylative elongation of carbon molecules of varying chain lengths and functional groups with only four core enzymes and no ATP requirement. Here, we describe recent developments in rBOX pathway engineering to produce alcohols and carboxylic acids with diverse functional groups, along with other commercially important molecules such as polyketides. We discuss the application of rBOX beyond the pathway itself by its interfacing with various carbon-utilization pathways and deployment in different organisms, which allows feedstock diversification from sugars to glycerol, carbon dioxide, methane, and other substrates.

## Graphical Abstract

**Graphical Abstract:**
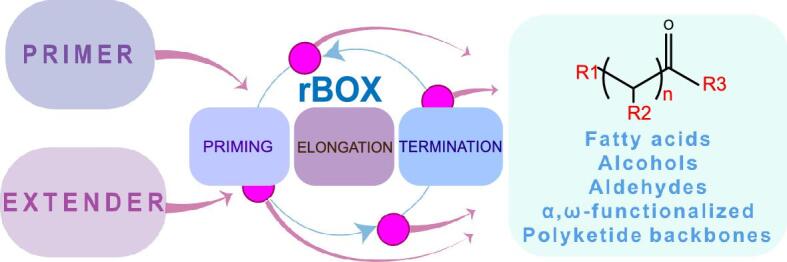
Reverse β-oxidation (rBOX) pathway allows efficient production of diverse bioproducts in a modular, controllable way.

## Introduction

Living systems can perform complex chemical reactions, a property that can be exploited for the synthesis of industrial products from renewable resources. Often the products that present commercial interest are higher chain length compounds synthesized from smaller (two to three carbon atom) precursor metabolites through carbon chain elongation pathways. Among these are fatty acids and alcohols (Cho et al., 2020), aldehydes (Kunjapur & Prather, 2015), hydrocarbons (Kang & Nielsen, 2017), isoprenoids (Lange et al., 2000), polyketides (Cai & Zhang, 2018), and other molecules that represent valuable fuels and commodity and fine chemicals. At its core, carbon chain elongation requires condensation reactions often carried out through the ketoacid pathway (Atsumi et al., 2008; Marcheschi et al., 2012), and fatty acid (Lennen & Pfleger, 2013; Marella et al., 2018), polyketide (Cai & Zhang, 2018; Staunton & Weissman, 2001; Zhou et al., 2020), and isoprenoid (Lange et al., 2000) biosynthesis. These natural pathways have inherent limitations to the product yield due to inefficient carbon and energy utilization, low flux, precursor competition with other essential metabolic pathways, and complex intrinsic regulation.

The reverse β-oxidation (rBOX) pathway circumvents many of these challenges using non-decarboxylative Claisen condensation reactions catalyzed by 3-ketoacyl-CoA thiolases (hereafter referred to as thiolases) to catalyze the condensation of acyl-CoA substrates and provide a carbon- and energy-efficient way to manufacture important molecules of commercial interest. The iterative nature of rBOX pathways makes them amenable to synthesizing an array of different chain length molecules, from C3 up to C16 (and theoretically beyond) (Cintolesi et al., 2014; Mehrer et al., 2018; Vick et al., 2015), while the choice of the termination pathway allows to tailor the product chain length and class. The most accessible product classes of rBOX pathways are alcohols and carboxylic acids; however, rBOX can be engineered to incorporate functionalized primer and extender units, as well as to incorporate novel biochemistries. This expanded functionality makes rBOX capable of synthesizing a wide range of products, such as α,ω-dicarboxylic acids and diols, ω-hydroxyacids and other functionalized fatty acid derivatives, β-hydroxy-ω-lactones, methyl alcohols, and others (Cheong et al., 2016). Furthermore, elements of the rBOX carbon chain elongation pathway can be combined with other biochemistries to create new synthetic pathways for the efficient production of more complex molecules such as polyketides (Tan et al., 2020).

This review will focus on advances in expanding the functionality of rBOX-based products. We will cover the basic principles for design of rBOX pathways, and strategies for diversifying the array of products within the established rBOX framework, as well as repurposing of this platform via integration with other metabolic pathways, creating novel synthetic routes for production of diverse molecule classes. We will also provide a future perspective on the rBOX architecture in the context of genome and protein engineering, dynamic and orthogonal regulation, and its transferability to other organisms.

## Concept and Reactions of the rBOX Pathway

As the name implies, the rBOX function relies on reversing the direction of the β-oxidation cycle. The native β-oxidation can be functionally reversed by circumventing the regulatory system of the fatty acid degradation pathway and generating a thermodynamic pull in the direction of chain elongation. The initial rBOX system engineering accomplished constitutive expression of the β-oxidation system through introduction of *fadR* and *atoC(c)* mutations, deletion of the *arcA* gene, and circumventing the carbon catabolite repression with a cAMP-independent crp* mutant (Dellomonaco et al., 2011). While effective, this system-level approach made it difficult to determine which of the many deregulated enzymes are responsible for product synthesis, which in turn limits efforts to fine-tune the synthesis of specific products and prevents the transfer of the engineered pathway to other organisms. Therefore, the system was subsequently redesigned using a bottom-up synthetic biology approach that allowed to isolate and elucidate the function of each individual component and create a modular rBOX framework that can be readily engineered and controlled (Clomburg et al., 2012).

The rBOX framework offers some unique advantages over other biosynthetic pathways. Compared with fatty acid and polyketide biosynthesis pathways, it circumvents the loss of CO_2_, increasing carbon yield and efficiency. Moreover, the rBOX cycle reactions do not require ATP, contributing to its energy efficiency. There are no inherent limitations to flux, as acetyl-CoA is a common precursor, and allows utilization of different carbon sources. rBOX also avoids complex regulatory mechanisms associated with the generation and/or utilization of malonyl-CoA/ACP. Each cycle requires two reducing equivalents in the form of NADH, which alleviates the need for supply of NADPH through the pentose phosphate pathway or other means. The biochemistry of the pathway enzymes allows for flexible precursor input and a diverse range of exit points, providing excellent flexibility and combinatorial capabilities to produce a wide range of products.

## Modular Framework for rBOX Pathways

**Fig. 1:**
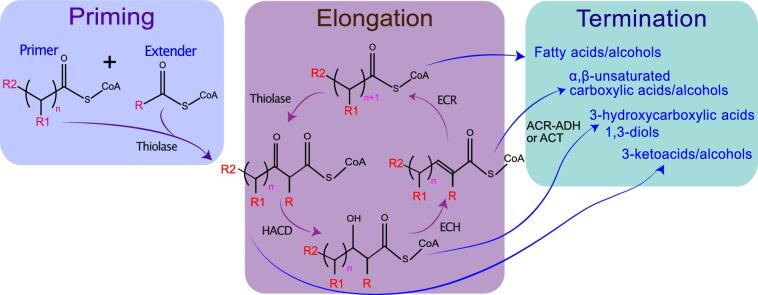
Modular structure of rBOX pathways and its use for production of alcohols and carboxylic acids with various functionalities. The three boxes represent conceptually discrete modules of the rBOX pathway, although they may share enzymes and substrates. HACD, hydroxyacyl-CoA dehydrogenase; ECH, enoyl-CoA hydratase; ECR, enoyl-CoA reductase; ACR-ADH, acyl-CoA reductase–alcohol/aldehyde dehydrogenase for the alcohol termination pathway; ACT, acetyl-CoA transferase (thioesterase) for the carboxylic acid termination pathway.

The general framework of rBOX includes functional modules for priming, elongation, and termination, with each cycle adding two carbons to the growing chain (Fig. 1). The priming module initiates the cycle by combining one primer and extender unit in a non-decarboxylative Claisen condensation reaction catalyzed by a 3-ketoacyl-CoA thiolase (Liu et al., 2020). During the reaction, a new carbon–carbon bond is formed between an acyl-CoA primer and an acetyl-CoA extender unit to form a β-keto ester. The standard primer and extender units of the rBOX pathway are two acetyl-CoA molecules; however, native and engineered thiolases can accept different types of functionalized acyl-CoAs as both primers and extenders (Bonk et al., 2018; Cheong et al., 2016; Kallscheuer et al., 2017; Liu et al., 2020). The thiolase-catalyzed reaction also acts as one of the control points for specifying the desired product by limiting the chain length based on its substrate specificity (Bonk et al., 2018; Dekishima et al., 2011; Kim & Gonzalez, 2018; Kim et al., 2015). The reversible thiolase condensation reaction is also a potential pathway bottleneck due to unfavorable thermodynamics (Kim, Cheong, Chou et al., 2016). Therefore, to drive the reaction in the desired direction of condensation, the levels of intracellular acetyl-CoA should be high and 3-ketoacyl-CoA low.

The elongation module consists of the four ‘core’ rBOX enzymes that perform the four steps of the elongation cycle. The first of these steps is also catalyzed by a 3-ketoacyl-CoA thiolase, which performs the same condensation reaction as the priming step but for subsequent cycles of elongation. The contextual distinction here lies in the difference of the priming and elongation units, which allows us to incorporate different functional groups into the growing molecule. By using thiolases with unique substrate specificity, it is possible to segregate these two reactions and use primer and extender units with different functionalizations (Cheong et al., 2016). The subsequent β-reduction steps of the rBOX cycle are catalyzed by hydroxyacyl-CoA dehydrogenase (HACD), enoyl-CoA hydratase (ECH), and enoyl-CoA reductase (ECR). These reactions generate an acyl-CoA intermediate two carbons longer than the initial primer, which can enter subsequent cycles of elongation. Different elongation enzyme variants possess different substrate length specificities, which can be exploited to customize the product chain length (Machado et al., 2012). It has also been shown that the combination of type II fatty acid biosynthesis enzymes and thiolases supports a functional rBOX (Clomburg et al., 2018). Replacing the β-oxidation enzymes HACD, ECH, and ECR with the equivalent enzymes from the bacterial type II fatty acid biosynthesis (FAB II) system (FabG, FabZ, and FabI) allows us to exploit the promiscuity of FAB enzymes, as they not only accept the ACP but also the CoA thioesters. The ability of some β-reduction enzymes to accept functionalized substrates makes it possible to create small molecules that were previously inaccessible by native pathways.

The exit from the cycle is facilitated by various termination enzymes, such as thioesterases, acyl-CoA reductases (ACRs), and aldehyde dehydrogenases, which specify the resulting product. In terms of termination module, fatty acid formation by thioesterases may be considered the ‘standard’ pathway for exit from the rBOX cycle. Thioesterases are ubiquitous and abundant, producing a range of carboxylic acids of different lengths (C4–C14; Clomburg et al., 2012; Vick et al., 2015). This presents a problem for targeting higher molecular weight products or producing other product classes, such as alcohols, as native thioesterases can compete with ACRs/alcohol dehydrogenases for flux. However, several strategies have been successfully employed to customize the product length and type. Kim et al. (2015) achieved high-yield production of medium-chain alcohols through deletion of native *Escherichia coli* thioesterases *yciA, ybgC, ydiI, tesA, fadM*, and *tesB.* This combined with overexpression of a selection of acyl-CoA reductase (ACR) variants from different organisms showed that rBOX strains are capable of producing alcohols in the C6–C10 range (Kim et al., 2015) (Table 1).

**Table 1.** Selected Examples of Products Synthesized Using rBOX Platforms

				Enzymes			
Product class	Carbon source	Product	Titer	Activation/priming	Elongation	Termination	Reference
Carboxylic acids	Glycerol	Butyrate	3.4 g/l	AtoB	FadB, egTER/FabI	Endogenous thioesterases	Clomburg et al. (2012)
	Glycerol	C6–C10 mixture	1.3 g/l	BktB	BktB, FadB, egTER	TesA	Kim et al. (2015)
	Glycerol	Decanoic acid	2.1 g/l	BktB	BktB, FadB, egTER	FadM	Kim & Gonzalez (2018)
	Glycerol	Valerate	∼398 mg/l	mePCT, BktB	TdTER,hbd, crt	Endogenous thioesterases	Tseng et al. (2010)
	Propionate and glucose	Valerate	1.425 g/l	mePct, BktB	PhaB, PhaJ4, tdTER	Fs2108	McMahon & Prather (2014)
	Glycerol	C6–C10 fatty acids	15.67 g/l (bioreactor)	BktB	BktB, fadB, egTER	YdiI	Wu et al. (2019)
	Glucose	Octanoic acid	0.74 g/l	PaaJ9	FadJ, tdTER	Thioesterase from *Anaerococcus tetradium*	Wang et al. (2021)
	Acetate and lactate	Butyrate	∼200 mM carbon	Endogenous rBOX enzymes from *Clostridium*			Detman et al. (2019)
	Lactate	*n*-Caproate	18 mol%				Liu et al. (2020)
	Methanol and propionate	*n*-Valerate	42.8 mM				de Smit et al. (2019)
	Methane	Butyrate	0.08 g/l	AtoB	Hbd, Crt, endogenous Ter	YdiI	Garg et al. (2018)
Dicarboxylic acids	Glucose	Adipic acid	639 μg/l	PaaJ	PaaH1, Ech, egTER	Ptb and Buk1	Yu et al. (2014)
	Glycerol	Adipic acid	2.5 g/l (bioreactor)	Cat1, PaaJ	PaaH, PaaF, tdTER	Acot8	Cheong et al. (2016)
	Glycerol	Glutarate	36.5 mmol/l	Tfu_0875	Tfu_2399, Tfu_0067, Tfu_1647	Tfu_2576-7	Zhao et al. (2018)
	Glycerol	C6–C10 dicarboxylic acids	0.5 g/l	BktB	BktB, FadB and egTer	YdiI, AlkBGT, ChnD, and ChnE	Clomburg et al. (2015)
ω-Hydroxyacids	Glycerol	C6–10 ω-hydroxyacids	> 0.8 g/l			YdiI, AlkBGT	
Alcohols	Glycerol	C6–C10 mixture	0.3 g/l	AtoB and FadA	AtoB, fadA, FadB, egTER	*M. aquaeoli* Maqu2507 ACR	Kim et al. (2015)
	Glucose	C4–C16 mixture	1.8 g/l (anaerobic)	*Vibrio fischeri* FadA	*Vibrio fischeri* FadAB, tdTER	*Marinobacter aquaeoli* ACR	Mehrer et al. (2018)
	Propionate and glucose	Pentanol	358 mg/l	BktB	Hbd, Crt, tdTER	*C. acetobutylicum* AdhE	Tseng & Prather (2012)
	Syngas	Hexanol	2.4 g/l (anaerobic)	Endogenous Thl	Endogenous Hbd, Crt, Bcd, EtfAB	Endogenous AdhE	Kottenhahn et al. (2021)
Alkanes	Glucose/glycerol	C3–C10 alkanes	0.2–4.3 mg/l	*Cupriavidus necator* BktB/*Clostridium acetobutylicum* thI	BktB, PhaB, PhaJ, tdTer	Endogenous thioesterases, *Prochlorococcus marinus* PMT1231, *Nocardia iowensis* CAR, and *Nostoc punctiforme* AD	Sheppard et al. (2016)
Methyl ketones	Glucose	2-Pentanone	0.24 g/l	BktB	Hbd, Crt and tdTer	PcaIJ from *P. putida, Reut_1331_1332*, and Adc from *C. acetobutylicum*	Lan et al. (2013)
	Glucose	Acetone	0.7 g/l				
3-Hydroxy-carboxylic acids	Glucose	(*R*)-3-Hydroxybutyrate	2.92 g/l	PhaA	PhaB	TesB	Tseng et al. (2009)
	Glucose	(*S*)-3-Hydroxybutyrate	2.08 g/l	Thl	Hbd		
	Butyrate	3-Hydroxyhexanoate	22.5 mg/l	Pct, BktB	BktB, PhaB	TesB	Martin et al. (2013)
	Glycerol	(*S*)-3-Hydroxybutyric acid	2.5 g/l	AtoB	FadB	Endogenous thiosterases	Clomburg et al. (2012)
	Glucose	(*S*)-3-Hydroxyvalerate	0.31 g/l	BktB, Ptb-Buk	Hbd	TesB	Tseng et al. (2010)
		(*R*)-3-Hydroxyvalerate	0.50 g/l		PhaB		
	Propionate and glucose	3-Hydroxyvalerate	2.16 g/l	mePct, BktB	PhaB	TesB	McMahon & Prather (2014)
	Glucose	3-Hydroxyoctanoate and 3-hydroxydecanoate	3.6 g/l	PaaJ9	FadJ, tdTER	Thioesterase from *Anaerococcus tetradium*	Wang et al. (2021)
	Syngas	(*S*)-3-Hydroxybutyric acid	14.63 g/l (anaerobic bioreactor)	Thl	Hbd	Native *C. autoethanogenum* thioesterase	Karim et al. (2020)
1,3-Diols		1,3-Butanediol	0.5 g/l (anaerobic bioreactor)			Native *C. autoethanogenum* aldehyde:ferredoxin oxidoreductase (AOR) and Adh	
	Glucose	1,3-Butanediol	27 mg/l	AtoB	FadB	MphF, FucO, or YqhD	Gulevich et al. (2016)
α,β-Unsaturated carboxylic acids	Glycerol	Crotonic acid	3.2 g/l (bioreactor)	BktB	FadB	YdiI	Kim, Cheong, & Gonzalez (2016)
		2-Hexenoic, 2-octenoic, and 2-decenoic acid	189 mg/l		BktB, FadB, egTER		
	Propionate and glucose	*Trans*-2-pentenoate	695 mg/l	mePCT, BktB	PhaB, PhaJ4	YdiI	McMahon & Prather (2014)
	Isobutyrate and glucose	4-Methylvalerate	570 mg/l		PhaB, PhaJ4, tdTER	Fs2108	
	Methane	Crotonic acid	0.06 g/l	AtoB	Hbd, Crt	YdiI	Garg et al. (2018)
Branched-chain products	Propionate and glycerol	Tiglic acid	3.9 g/l	mePCT, FadAx	FadB2x, FadB1x	YdiI	Cheong et al. (2016)
	Isobutyrate and glycerol	4-Methylpentanol	35 mg/l	Pct, BktB	FadB, egTER	Maqu2507	
	Isobutyrate	3-Hydroxy-4-methylvalerate	1.8 mg/l	mePCT, BktB	BktB, PhaB	TesB	Martin et al. (2013)
	Glycolate	3,4-Dihydroxybutyric acid	0.3 mg/l				
	Glucose and propionate	α-Methyl enoic acid	1120 mg/l	Pct, *Ascaris suum* Acat5	AsHadH, EcH	YdiI	Blaisse et al. (2017)
	Glucose	4-Methylpentanol	192 mg/l	*Rhodopseudomonas palustris* IbuA, *Cupriavidus necator* BktB	PhaB, PhaJ4, tdTER	Car (*N. iowensis*), ADH6 (*Leifsonia* sp)	Sheppard et al. (2014)
	Glycerol	4-Methylvalerate	34 mg/l	BktB	FabG, FabZ (R126W R121E), FabI	Endogenous thiosterases	Clomburg et al. (2018)
Polyketides	Glycerol and hexanoate	Olivetolic acid	80 mg/l	BktB	BktB, FadB, egTER	OLS and OAC	Tan et al. (2018)
	Glycerol	Triacetic acid lactone (TAL)	0.86 g/l	BktB	N/A	Spontaneous lactonization	Tan et al. (2020)
	Glycerol	Alkylresorcinolic acid (ORA) and orcinol	∼5 mg/l	BktB	N/A	*C. sativa* olivetolic acid cyclase (OAC)	Tan et al. (2020)
Polyester	Glucose and propionate	α-Methyl-branched PHA	18 mmol/l	AsAcat3, cpPCT	RephaB	CapPhaEC	Dong et al. (2019)
	Glycolate	PHA	1 wt%	mePCT, BktB	RephaB1	RephaC1	Insomphun et al. (2016)
	Methanol	PHA	5.4 mol% 3HV, 0.9% 3HHx	Emd, BktB	ReHbd, ReCrt2	AcPhaC_NSDG_	Orita et al. (2022)

## Overlapping Chain Length Specificity of Multiple Enzymes

Since rBOX is an iterative pathway and can generate an array of different chain-length products, one of the biggest challenges is tailoring the product length. As mentioned earlier, almost every rBOX enzyme represents a point of control that can either allow or restrict flux through the associated nodes. The ability to mix and match the specificity range of different rBOX modules allows for tailoring of the product length, as was demonstrated in the production of decanoic acid (Kim & Gonzalez, 2018). This was achieved by selecting rBOX elongation enzymes that support the synthesis of acyl-CoA molecules of up to 10 carbons (using a broad-range BktB thiolase from *Cupriavidus necator* [previously known as *Ralstonia Eutropha*] [Chen et al., 2015; Slater et al., 1998] and egTER [ECR from *Euglena gracilis*]) and a termination module (FadM thioesterase) that exhibited high activity toward decanoyl-CoA and longer chain acyl-CoAs. The final engineered strain produced 2.1 g/l of decanoic acid as the primary fermentation product with a yield of 0.1 g/g glycerol (Table 1).

## Pathway Termination at Different Nodes

**Fig. 2:**
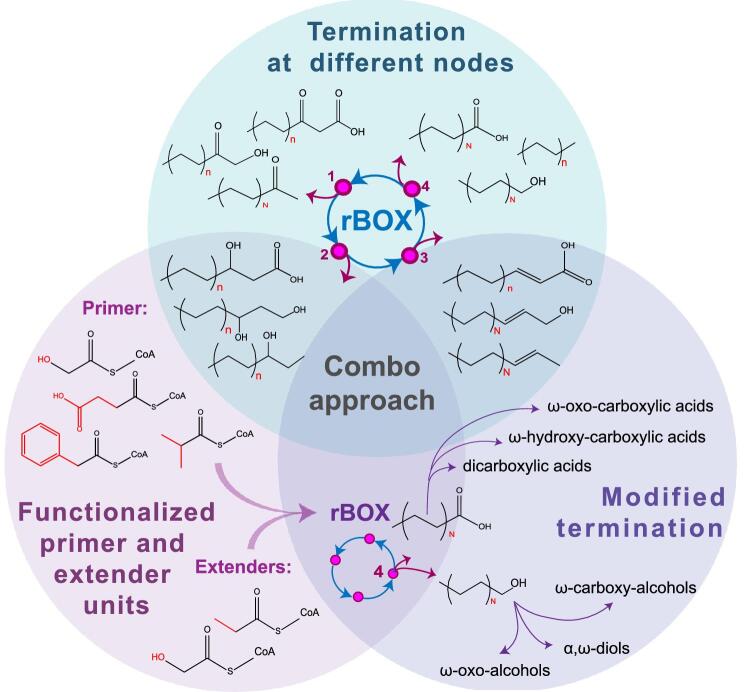
Diversifying rBOX product classes through different strategies: (1) termination at different cycle nodes; (2) using alternative primers (glycolyl-CoA, succinyl-CoA, phenylacetyl-CoA, isopropyl-CoA, and others not shown) and extender units (glycolyl-CoA, propionyl-CoA); and (3) additional enzymatic modification at the ω group. These approaches can also be combined to further diversify the product profile.

An alternative approach to customizing rBOX products is cycle termination from different nodes, which results in the formation of alternative product classes with different degrees of reduction and functionalization (Fig. 2). For example, termination at the first intermediate in the rBOX cycle (3-ketoacyl-CoA), followed by a CoA group transfer and decarboxylation, can produce methyl ketones. This was shown by Lan et al. 2013 using a combination of BktB (thiolase), Hbd (HACD), Crt (ECH), and Ter (ECR) and a termination module consisting of 3-oxoadipate CoA-succinyl transferase PcaIJ from *Pseudomonas putida*, which acts on 3-ketohexanoyl-CoA, and an acetoacetate decarboxylase (Adc). Two rounds of rBOX followed by transferase and decarboxylase steps yielded 0.24 g/l of 2-pentanone. Similarly, using a CoA transferase Reut_1331 and Reut_1332 led to production of 0.7 g/l of acetone (Lan et al., 2013) (Table 1).

Cycle termination at the 3-hydroxyacyl-CoA node results in a number of important products, including 3-hydroxycarboxylic acids and 1,3-diols. For example, an incomplete rBOX cycle terminating after the initial condensation (by thiolases BktB, Thl, or PhaA) and dehydrogenation (by HACDs Hbd, or PhaB) steps followed by hydrolysis of the CoA thioester by a thioesterase resulted in the production of enantiomerically pure (*R*)-3-hydroxybutyrate and (*S*)-3-hydroxybutyrate to concentrations of 2.92 and 2.08 g/l, respectively (Tseng et al., 2009). This route was subsequently modified to produce 22.5 ± 5.9 mg/l 3-hydroxyhexanoate by supplying precursor substrate butyrate and expressing a broad substrate specificity CoA activation enzyme propionyl-CoA transferase (Pct) from *Megasphaera elsdenii* (Martin et al., 2013). Clomburg et al. (2012) also reported 2.5 g/l of (*S*)-3-hydroxybutyric acid (using glycerol as a carbon source) by overexpression of *atoB* (thiolase) and *fadB* (HACD) in a mixed-acid fermentation-deficient strain, with a 0.29 g/g yield (Clomburg et al., 2012). Termination at the 3-hydroxyacyl-CoA node can result in the production of 1,3-butanediol (1,3-BDO). Gulevich reported 27 mg/l of 1,3-BDO using aldehyde dehydrogenase (*mhpF*) from *E. coli* to reduce 3-hydroxybutyryl-CoA into 3-hydroxybutyaldehyde, followed by the use of alcohol dehydrogenase (*fucO* or *yqhD*) to yield 1,3-BDO (Gulevich et al., 2016) (Table 1).

The third node of the rBOX pathway, trans-enoyl-CoA, can be used as an exit point from the cycle generating α,β-unsaturated carboxylic acids or to regenerate the acyl-CoA intermediate that enters into the next round of rBOX. A native *E. coli* thioesterase YdiI was used to convert trans-enoyl-CoA to the corresponding α,β-unsaturated carboxylic acids. Its expression in combination with a partial rBOX cycle overexpressing thiolase BktB and native FadB (performing dual HACD and ECH) resulted in crotonic acid production at titers reaching 3.2 g/l with a yield of 0.12 g crotonic acid/g glycerol. The omission of ECR enzyme prevented competition for substrate for crotonic acid production. Alternatively, including egTER (ECR) led to production of 2-hexenoic acid, 2-octenoic acid, and 2-decenoic acid at a titer of 0.2 g/l, demonstrating for the first time microbial production of unsaturated carboxylic acids (Kim, Cheong, & Gonzalez, 2016) (Table 1).

## Using Alternative Primer and Extender Units

The standard rBOX pathway using acetyl-CoA as both primer and extender units results in even-numbered carbon chain products. This apparent limitation can be overcome by removing a carbon as a part of the termination pathway (Lan et al., 2013) or by supplying odd-chain primer molecules (Fig. 2). For example, Tseng et al. (2010) engineered a pathway for production of chiral 3-hydroxyvalerate (3HV) through condensation of propionyl-CoA and acetyl-CoA (Tseng et al., 2010). They showed that propionate could be supplied exogenously and activated by a Ptb–Buk complex or endogenously produced from glucose or glycerol via overexpressing the threonine biosynthesis pathway. Propionyl-CoA was fed into a one-turn incomplete rBOX cycle expressing thiolase BktB (which was demonstrated to accept C3 substrates), PhaB, or Hbd (HACD) and TesB to produce 0.31 g/l and 0.50 g/l of (*S*)-3HV and (*R*)-3HV from glucose (Table 1).

This approach was further expanded to produce odd-chain alcohols (pentanol) (Tseng & Prather, 2012) and C3–C10 alkanes by including a complete list of rBOX cycle enzymes (thiolase BktB from *C. necator* or ThlA from *Clostridium acetobutylicum*, reductase PhaB, and dehydratase PhaJ from *C. necator* and ECR Ter from *Treponema denticola*) and using a termination module consisting of endogenous thioesterases, a broad-specificity carboxylic acid reductase (CAR), and aldehyde decarbonylase (AD) to convert C*n* fatty acids into corresponding C(*n* −* 1*) alkanes via a C*n* fatty aldehyde intermediate (Sheppard et al., 2016). Another example of utilizing propionate as a building block activated by *Megaphaera elsdenii* PCT (mePCT) and with AtoB (thiolase), FadBA (thiolase, HACD, ECH), and FabI (ECR) as priming and elongation modules produced odd-chain carboxylic acids ranging from C5 to C11 at titers from 187 to 30.9 mg/l, respectively (Vick et al., 2015) (Table 1). This study also showed that endogenous *E. coli* enoyl-ACP reductase FabI can act as the ECR-supporting rBOX function.

Besides straight-chain aliphatic compounds, rBOX can also be utilized for production of branched or other functionalized compounds by feeding primer and/or extender units with various functional groups. While the use of propionyl-CoA as primer leads to the synthesis of straight, odd-chain products, the use of it as extender unit it yields α-branched products such as 2-methyl-2-enoic acids and 2-methyl acids. Up to 3.9 g/l tiglic acid was produced in a controlled bioreactor through an rBOX pathway consisting of FadAx, FadB1x, and FadB2x along with the mePCT transferase for activation of supplemented precursor propionate (Cheong et al., 2016). In a separate study, ketoreductase was identified to be the key driver for selectivity, forming predominantly α-branched products even when paired with a thiolase that highly prefers unbranched products. Leveraging the specificity of this ketoreductase from *Ascaris suum* allowed production of chiral 2-methyl-3-hydroxy acids (1.1 ± 0.2 g/l) or branched enoic acids (1.12 ± 0.06 g/l) at 44% and 87% yields of fed propionate, respectively (Blaisse et al., 2017). Synthesis of a ω-1-branched product is also possible by using a branched acyl-CoA primer such as isobutyryl-CoA. For example, Martin et al. (2013) reported production of 3-hydroxy-4-methylvalerate through an rBOX pathway consisting of BktB and PhaB along with the mePCT for activation of isobutyrate (Martin et al., 2013). Similarly, Sheppard et al. (2014) produced 4-methylpentanol at 192 ± 23 mg/l (Sheppard et al., 2014). Clomburg et al. (2018) reported that *E. coli* type II fatty acid biosynthesis enzymes (FabG, FabZ, and FabI) can produce the ω-1-branched product 4-methylvalerate through rBOX at higher titer than rBOX enzymes (FadB, egTER, and PhaJ) (Clomburg et al., 2018) (Table 1).

Another variant of the rBOX pathway composed of PaaJ (thiolase), PaaH1 or Hbd (HACD), Crt or Ech (ECH), and *E. gracilis* Ter (TER) can use ω-carboxylated succinyl-CoA as the primer to produce adipic acid, an important bulk chemical used as the monomer of nylon-6,6. Using Ptb (phosphate butyryltransferase) and Buk1 (butyryl kinase) as a termination module allowed the biosynthesis of adipic acid from glucose in *E. coli* (Yu et al., 2014). Similarly, natively produced malonyl-CoA can be used as a primer to form an odd-chain dicarboxylate, glutarate, expressing rBOX pathway genes from *Thermobifida fusca* specific for dicarboxylate synthesis (Zhao et al., 2018). A more comprehensive study reported a modular rBOX approach for using different ω- and ω-1-functionalized primers and α-functionalized extender units in combination with various termination pathways (Cheong et al., 2016). It demonstrated using 7 different primers and 3 extender units to synthesize 18 functionalized compounds belonging to 10 different classes: phenylalkanoic, dicarboxylic, ω-hydroxy, 4-oxo, 4-methyl, 2-methyl, 2-methyl-2-enolic and 2,3-dihydroxy acids, β-hydroxy-ω-lactones, and 4-methyl alcohols (Cheong et al., 2016).

## Termination Through Alpha and Omega Oxidation

Additional modifications to the rBOX termination module allow us to further expand its functionality (Fig. 2). In one example of this approach, the core rBOX modules (BktB/thiolase, FadB/HACD-ECH and egTer/TER), along with thioesterase YdiI for cycle termination, were combined with ω-oxidation pathways by expressing ω-hydroxylase (AlkBGT), alcohol dehydrogenase (ChnD or YjgB), and aldehyde dehydrogenase (ChnE) to convert carboxylic acids to ω-hydroxyacids and/or dicarboxylic acids (Clomburg et al., 2015). AlkBGT expression resulted in production of >0.8 g/l of C_6_, C_8_, and C_10_ ω-hydroxyacids from glycerol (Clomburg et al., 2015). In a second ω-oxidation step, overexpression of alcohol dehydrogenase ChnD and aldehyde dehydrogenase ChnE further led to production of nearly 0.5 g/l of C_6_–C_10_ diacids (Table 1).

The rBOX platform can also be extended by α-oxidation. This pathway is characterized by the shortening of fatty acids by one carbon, resulting in odd-chain fatty aldehydes, which can be further converted to other products. Although this pathway has not been demonstrated with rBOX, it has been applied to modification of fatty acids produced through the fatty acid biosynthesis pathway. For example, Kaehne et al. (2011) engineered *E. coli* expressing α-dioxygenase to convert C_10_–C_18_ fatty acids into shortened fatty aldehydes (Kaehne et al., 2011). Cao et al. (2015) went further by engineering *E. coli* to use α-dioxygenase in combination with an aldehyde reductase to produce ∼100 mg/l odd-chain C_11_–C_15_ fatty alcohols from glycerol (Cao et al., 2015).

## Combination Approach

Some of the examples listed earlier involve a combination of different approaches to synthesize specific rBOX-derived products (Fig. 2). For example, Martin et al. (2013) used functionalized primers (like glycolyl-CoA) and a termination at the 3-hydroxyacyl-CoA node to produce diverse dihydroxyacids, such as 3,4- dihydroxybutyric acid (3,4-DHBA). The system was supplemented with glycolate, which was converted to glycolyl-CoA by a broad substrate specificity mePCT and a propionyl-CoA synthetase (PrpE) from *Salmonella typhimurium* LT2, in addition to Ptb–Buk. This approach allowed synthesis of five novel products: 3,4-DHBA, 3HBL, 2,3-dihydroxybutyric acid (2,3-DHBA), 3-hydroxyhexanoic acid (3HH), and 3-hydroxy-4-methylvaleric acid (3H4MV) (Martin et al., 2013). Another example of a combination approach is using an alternative termination pathway expressing carboxylic acid reductase (Car) from *Nocardia iowensis* to convert fatty acids to alcohols. This was demonstrated by producing 4-methyl-pentanol from endogenously generated isobutyryl-CoA as a primer (Sheppard et al., 2014). Combination of biotic and abiotic catalysis provides novel opportunities for product diversification. Wang et al. (2021) utilize 3-hydroxyacids produced through rBOX as precursor to produce olefins using heterogenous Lewis acidic catalysts. This abiotic approach enables production of olefins from fatty acids without any additional redox input (Wang et al., 2021).

## Interfacing rBOX With Various Metabolic Pathways and Its Deployment in Different Organisms

**Fig. 3:**
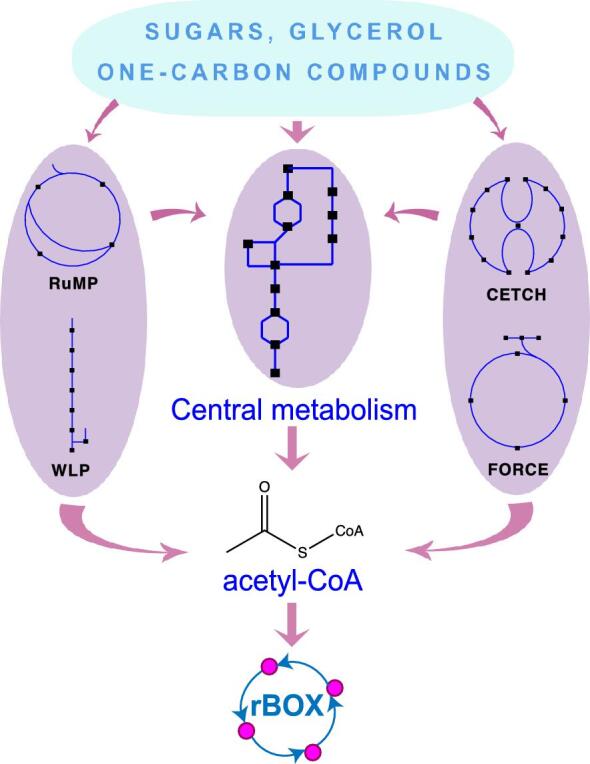
Interfacing central metabolism and upstream substrate utilization pathways with the rBOX platform. Methanotrophs and methylotrophs harboring ribulose monophosphate (RuMP) can assimilate methane, methanol, and/or formaldehyde into central metabolites, which can then be converted to acetyl-CoA. The Wood–Ljungdahl pathway (WLP) converts CO_2_ or CO into acetyl-CoA. The synthetic CO_2_-fixing crotonyl-CoA/ethylmalony-CoA/hydroxybutyryl-CoA (CETCH) cycle (Schwander et al., 2016) operates with various CoA thioesters as pathway intermediates, which can be directly utilized as acyl-CoA primers or extenders for rBOX or further converted into acetyl-CoA via central metabolism. Formyl-CoA Elongation (FORCE) pathways (Chou et al., 2021) can utilize various one-carbon (C1) substrates to produce diverse acyl-CoAs, including acetyl-CoA.

The ubiquitous nature of β-oxidation and the building block acetyl-CoA allows interfacing with other metabolisms and microbial hosts. The rBOX pathway has been successfully implemented in various industrial chassis such as *Saccharomyces cerevisiae* (Lian & Zhao, 2015) and *Corynebacterium glutamicum* (Shin et al., 2021), illustrating the cross-platform capabilities of the pathway. Moreover, native rBOX capabilities of *Clostridium* species have been leveraged in the context of expanding substrate diversity. For example, lactate (Liu et al., 2020), acetate and lactate (Detman et al., 2019), and methanol and propionate (de Smit et al., 2019) have been utilized as feedstock to produce butyrate, caproate and isobutyrate, and *n*-valerate, respectively. rBOX can also be implemented in pathways and organisms that utilize one-carbon (C1) molecules such as CO_2_ and methane to contribute to global carbon mitigation efforts. Various native and synthetic CO_2_-fixation pathways, such as the Wood–Ljungdahl pathway (WLP) (Ragsdale, 2008), reductive tricarboxylic acid (TCA) cycle (rTCA) (Berg, 2011), and crotonyl-CoA/ethylmalony-CoA/hydroxybutyryl-CoA (CETCH) cycle (Schwander et al., 2016) either produce acetyl-CoA as product (WLP and rTCA) or utilize various acyl-CoAs as intermediates (CETCH), which can be directed into rBOX as primer or extender units (Fig. 3). As an example, production of up to 2.4 g/l hexanol from syngas through WLP followed by rBOX has been demonstrated in *Clostridium carboxidivorans* P7 (Kottenhahn et al., 2021) (Table 1). Other C1 utilization pathways that do not involve CoA thioesters as direct intermediate or product can still be rewired into rBOX via central metabolism (Fig. 3). For example, *Methylomicrobium buryatense* 5GB1C, a methanotroph that utilizes methane via the ribulose monophosphate (RuMP) cycle, was engineered to produce 4-carbon carboxylic acids via rBOX by directing metabolic flux toward acetyl-CoA generation (Garg et al., 2018). In another study, production of polyhydroxyalkanoates (PHAs) from methanol through rewiring the native serine cycle and ethylmalonyl-CoA pathway in conjunction with heterologously expressed rBOX pathway enzymes was demonstrated in *Methylorubrum extorquens* AM1 as host chassis (Orita et al., 2022). Other native and synthetic C1 utilization pathways can either directly generate acyl-CoAs or produce precursor metabolites that connect to central metabolism to generate acetyl-CoA. For example, the recently developed formyl-CoA elongation (FORCE) pathways (Chou et al., 2021) convert C1 substrates in the form of formyl-CoA to C2 and longer-chain 2-hydroxyacyl-CoAs, which can also be further reduced to acyl-CoAs, and serve as both primer and extender units for the rBOX pathway (Fig. 3).

Diversifying substrates for primer and extender units or transferring rBOX into a different organism often requires extensive enzyme and metabolic flux optimization. Recent studies show that substrate specificity and activity of rBOX pathway enzymes can be expanded and optimized via bioprospecting (Mehrer et al., 2018; Tan et al., 2020; Wang et al., 2021) and protein engineering (Blaisse et al., 2018; Clomburg et al., 2018). Incorporating the rBOX pathway in non-model organisms, such as *Clostridium*, is more challenging than in common industrial hosts, such as *E. coli, S. cerevisiae*, and *C. glutamicum*. A platform developed by Karim et al., named *in vitro* prototyping and rapid optimization of biosynthetic enzymes (iPROBE), addresses this problem by using the cell-free system for rapid optimization of the pathway enzymes and expression levels and showing that it can be transferred to *in vivo* (*Clostridium*) with a strong correlation (Karim et al., 2020). Optimization of the one-turn rBOX pathway for 3-hydroxybutyrate and *n*-butanol production has been demonstrated by high-throughput screening of more than 200 pathway combinations, which were then engineered into *Clostridium autoethanogenum* to demonstrate 20-fold increase in 3-hydroxybutyrate titer to ∼15 g/l (Karim et al., 2020). iPROBE was further used to accelerate carbon-negative manufacturing of acetone and isopropanol in *C. autoethanogenum* (Liew et al., 2022).

## Products of rBOX as Building Blocks for Downstream Pathways

To further exploit the carbon and energy economy of the rBOX pathway, products derived from this platform could be utilized to feed into other metabolic routes. One example of using rBOX products as building blocks is the synthesis of bioderived polymers such as PHAs. Dong et al. (2019) used acetyl-CoA and propionyl-CoA to produce (3*R*)-hydroxyacyl-CoA which can be copolymerized by a PHA polymerase to ultimately form α-branched polyesters (Dong et al., 2019). In this study, the substrate selectivity of thiolase (AsAcat3) and HACD (RePhaB) plays a key role in controlling monomer availability for incorporation into the final copolymer by the PHA polymerase. Similarly, Insomphun et al. (2016) constructed an artificial pathway for the production of PHA containing a DHBA monomer by introducing the *pct* gene from *M. elsdenii*, along with the *bktB* (thiolase) and *PhaB1* (HACD) genes from *C. necator* (Insomphun et al., 2016). By feeding glycolate, the production of PHA with 1 mol % DHBA reached 1% of the dry cell weight (Table 1).

**Fig. 4:**
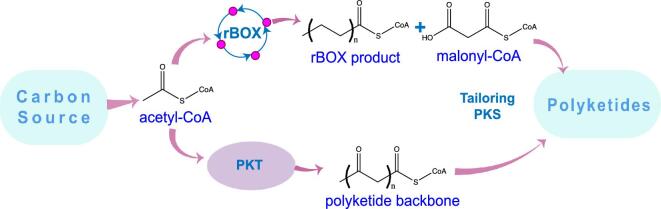
Integrating elements of the rBOX pathway into polyketide biosynthesis in one of two approaches: (1) through condensation of rBOX pathway products with malonyl-CoA using polyketide synthases (PKSs) or (2) through synthesis of polyketide backbones in a PKS-independent manner by using polyketoacyl-CoA thiolase (PKT).

Another example is polyketide biosynthesis (Fig. 4). Elements of rBOX have been successfully integrated into the polyketide synthesis framework to produce olivetolic acid (OLA), a plant secondary metabolite sourced from type III polyketide synthase (PKS). Here, the core rBOX modules were integrated into the chromosome (thiolase BktB, HACD-ECH FadB, and ECR egTER) for production of a hexanoate precursor, along with a native *E. coli* fatty acyl-CoA synthetase FadD to generate hexanoyl-CoA (Tan et al., 2018). The rBOX framework for precursor synthesis was combined with expression of PKS enzymes OLA synthase and OLA cyclase from *Cannabis sativa*, demonstrating for the first time the microbial synthesis of olivetolic acid from a single carbon source at 80 mg/l (Table 1).

Utilizing elements of the rBOX pathway in a novel way was explored by Tan et al. (2020) for production of polyketide backbones (Tan et al., 2020) (Fig. 4). The canonical polyketide biosynthesis pathway proceeds via PKSs, which catalyze iterative decarboxylative Claisen condensation using malonyl-CoA as the extender unit. However, Tan et al. (2020) discovered that certain thiolases, which they named polyketoacyl-CoA thiolases (PKTs), catalyze polyketide backbone formation via iterative non-decarboxylative Claisen condensations, hence offering a synthetic and efficient alternative to PKSs. Additionally, the basic catalytic mechanism and structural features enabling this previously unknown activity were elucidated. For the first time, synthesis of polyketide triacetic acid lactone (TAL) of up to 0.86 g/l was demonstrated only with the expression of a PKT (BktB) and in the absence of PKS (Table 1). It was further demonstrated that PKT can replace PKS in a higher number of iterations, showing alkylresorcinolic acid (ORA) and orcinol production from an *in situ* generated 3,5,7-trioxooctanoyl-CoA backbone (Tan et al., 2020).

The PKT-based framework was also used to demonstrate *in vitro* synthesis of 6-MSA and *m*-cresol (decarboxylated form of 6-MSA), which is normally catalyzed by a large, multimodular type I 6-MSA PKS. *In vitro* production was demonstrated using acetoacetyl-CoA primer and acetyl-CoA extender by combined expression of PKT BktB with the thioester hydrolase (TH) and ketoreductase (KR) domains from *Aspergillus terreus.* Additionally, BktB mutants were shown to accept butyryl-CoA primer and acetyl-CoA as the extender unit, further expanding the range of possible polyketide backbones that can be produced by exploiting the PKT activity of thiolases (Tan et al., 2020). Given the promiscuous nature of some thiolase variants, it is possible that they could be engineered to incorporate functionalized molecules into the polyketide backbone beyond the limited PKS building blocks (acetyl-CoA or propionyl-CoA as primers and malonyl-CoA or methylmalonyl-CoA extender units), creating new-to-nature chemistries.

## Optimization of Pathway Efficiency and Productivity

Optimization of the rBOX pathway involves strain engineering to improve acetyl-CoA availability and balance the redox cofactor, enzyme identification and engineering for better kinetic properties, and fermentation optimization for improved titer, rate, and yield. Initial rBOX strain engineering focused on eliminating alternative acetyl-CoA utilization pathways by deleting the mixed-acid fermentation (Kim, Cheong, Chou, et al., 2016). Additionally, deletion of endogenous thioesterases to prevent premature hydrolysis of CoA thioesters has been shown to improve product titers and yields (Kim et al., 2015). Alternatively, the same objective was achieved by downregulating fermentative pathway genes using CRISPRi and rewiring prematurely hydrolyzed acetate back to rBOX using acetyl-CoA synthetase (Wu et al., 2017). Moreover, incorporating a mutant pyruvate dehydrogenase (PDH) with improved activity under microaerobic conditions with high NADH/NAD^+^ ratio improved flux toward acetyl-CoA from central metabolism (Wu et al., 2019).

Identification of optimal pathway enzymes is key for improving specificity toward the desired product in an iterative pathway like rBOX. Research shows that thiolases and termination enzymes are two key nodes to control the chain length and functionality of the desired products. Thiolases and thioesterases with desired activity have been identified by molecular phylogenetic analysis (Mehrer et al., 2018; Tan et al., 2020; Wang et al., 2021) or constructed via protein engineering (Blaisse et al., 2018). Moreover, *in vitro* prototyping allows for high-throughput screening of a large number of enzyme variants like thioesterases to identify special functional group and chain length specificity (McMahon & Prather, 2014). Alternatively, substrate specificity of core rBOX enzymes like ketoreductases could influence specificity of upstream thiolase, leading to improved selectivity (Blaisse et al., 2017). Furthermore, optimization of relative expression levels is critical for debottlenecking the pathway flux, which could be facilitated by rapid *in vitro* prototyping (Karim et al., 2020) and independent gene expression control using orthogonal inducible promoters (Meyer et al., 2019) for *in vivo* implementation.

Since medium-to-long-chain carboxylic acids and alcohols are more reduced than sugars, NADH availability and sufficient NADH/NAD^+^ ratio are important for high productivity. Optimization of the oxygen transfer rate is one approach to address this. Maximizing flux toward rBOX while circumventing regulatory systems under the microaerobic condition was shown to be important in improving the product titer. For example, upregulating PDH activity under a high NADH/NAD^+^ ratio by utilizing mutant PDH and downregulating fermentative pathways through CRISPRi were crucial for improving mid-chain fatty acid titer under microaerobic condition (Wu et al., 2019). Another key consideration is addressing the product toxicity and volatility with fatty acids and alcohols of various chain lengths having distinctive toxic effects on *E. coli* (Wilbanks & Trinh, 2017). Two-phase fermentations with an organic phase serving as extraction solvent during the fermentation was shown as an effective approach to address this issue (Kim et al., 2015; Kottenhahn et al., 2021).

## Conclusions and Future Directions

The rBOX platform offers high energy and carbon efficiency compared with other pathways for carbon chain elongation and has the potential to be orthogonal to the host metabolism, thus minimizing unproductive crosstalk with life-sustaining reactions. In addition, rBOX operates at higher flux than other anabolic pathways and is modular, customizable, and programmable. The ability to use various primer and extender units, along with different termination mechanisms and the possibility of integrating the cycle products as intermediates for other pathways, makes rBOX a versatile platform for the synthesis of diverse product classes, including new-to-nature molecules. In addition, the ubiquitous nature of β-oxidation chemistries creates a potential for transferring these capabilities to other industrial organisms. Subsequently, we discuss some of the approaches that can be used to address remaining challenges, including the improvement of product titer, rate, and yield and the implementation of rBOX in other organisms.

One of the key considerations in tailoring rBOX to generate products of a specific chain length and functionality as well as in optimizing pathway flux to improve rate and titer is the identification of an enzyme candidate with optimal kinetic properties. Numerous rBOX enzymes have been characterized, with some having a narrow substrate range while others accepting a wide range of carbon chain lengths and functional groups. Recent developments in protein structure modeling tools such as AlphaFold (Jumper et al., 2021) provide a host of opportunities for protein structure analysis and engineering without the need for crystal structures. Learning from the structure analysis of the current database of rBOX enzymes aided by these tools can substantially enhance the knowledge of kinetic properties and the engineering of these enzymes for desired activity and specificity. In addition, low-cost protein engineering techniques in conjunction with high-throughput screening methods can reduce the time required for testing a large number of enzyme variants.

Fine-tuning the expression of enzymes to debottleneck pathway flux and maximize yield is another key consideration. Several dynamic and orthogonal regulation tools have been developed to help achieve this goal. Dynamic regulation of gene expression controlled by sensors such as light, pH, and metabolites has been shown to improve biosynthesis of diverse molecules by limiting biomass, by-products, and accumulation of toxic intermediates (Dinh & Prather, 2020; Shen et al., 2019). Moreover, decoupling growth and stationary phases via dynamic deregulation of key nodes in central metabolism during stationary phase can improve process robustness and scalability (Ye et al., 2021). In addition, the importance of relative enzyme availability for pathway optimization has been demonstrated both *in vivo* through orthogonal inducible promoter (Meyer et al., 2019) and *in vitro* through cell-free prototyping platforms (Karim et al., 2020). Altogether, these recently developed tools and techniques provide a great opportunity for improving the rBOX platform.

With increasing interest in using non-model organisms for direct utilization of greenhouse gases and organic wastes such as plastics to accomplish sustainability and combat climate change, it is imperative to develop host-agnostic, orthogonal platforms that can be transferred to different chassis organisms. rBOX is a great example of such platforms as it utilizes a universal metabolic precursor (acetyl-CoA) to produce a wide range of molecules with varying chain lengths and functionalities. Moreover, deletion of native β-oxidation genes as well as other competing pathways (e.g. thioesterases) for acetyl-CoA and other acyl-CoA pools can make rBOX orthogonal to central metabolism. Advances in genome engineering tools, such as CRISPR/Cas9, have made engineering of non-model organisms very tractable. It has already been demonstrated that organisms that do not produce a high acetyl-CoA pool (notably organisms that mainly utilize pyruvate decarboxylase, such as *S. cerevisiae*) can be engineered to do so as well (Meadows et al., 2016). The future of rBOX will expand not just on the product diversity but also on the substrate diversity for more sustainable biomanufacturing that can support a circular bioeconomy.
